# Mass spectra prediction with structural motif-based graph neural networks

**DOI:** 10.1038/s41598-024-51760-x

**Published:** 2024-01-16

**Authors:** Jiwon Park, Jeonghee Jo, Sungroh Yoon

**Affiliations:** 1 Interdisciplinary Program in Artificial Intelligence, Seoul National University, Seoul, 08826 Republic of Korea; 2 LG Chem, Seoul, 07795 Republic of Korea; 3 Center for Neuromorphic Engineering, Korea Institute of Science and Technology (KIST), Seoul, 02792 Republic of Korea; 4 Department of Electrical and Computer Engineering, Seoul National University, Seoul, 08826 Republic of Korea; 5 Artificial Intelligence Institute, Seoul National University, Seoul, 08826 Republic of Korea

**Keywords:** Chemistry, Engineering, Materials science, Mathematics and computing

## Abstract

Mass spectra, which are agglomerations of ionized fragments from targeted molecules, play a crucial role across various fields for the identification of molecular structures. A prevalent analysis method involves spectral library searches, where unknown spectra are cross-referenced with a database. The effectiveness of such search-based approaches, however, is restricted by the scope of the existing mass spectra database, underscoring the need to expand the database via mass spectra prediction. In this research, we propose the Motif-based Mass Spectrum prediction Network (MoMS-Net), a GNN-based architecture to predict the mass spectra pattern utilizing the structural motif information of the molecule. MoMS-Net considers both a molecule and its substructures as a graph form, which facilitates the incorporation of long-range dependencies while using less memory compared to the graph transformer model. We evaluated our model over various types of mass spectra and showed the validity and superiority over the conventional models.

## Introduction

Mass spectrometry (MS)^1,2^ is an indispensable analytical method for the identification of molecular structures in unknown samples^3–5^. In this technique, a molecule undergoes ionization, and its fragment ions are measured by a mass analyzer, which captures information regarding the mass-to-charge ratio (m/z). By analyzing the mass spectrum, which provides the m/z values and their relative intensities, it is possible to infer the molecular structure of the original chemical.

Modeling the fragmentation patterns of ionized molecules for analyzing the mass spectrum is challenging. While several domain knowledge-based rules can be useful for certain types of molecules, they are limited in the application of smaller fragments with diverse functional groups.

The interpretation of mass spectra typically relies on the public library search, by comparing the obtained spectra of an unknown target molecule with the large database of known molecules^6,7^. There exist various extensive mass spectra libraries available, such as the National Institute of Standards and Technology (NIST)^8^, Wiley^9^, and Mass Bank of North America (MoNA)^10^. However, the search-based method has a limitation of its accessibility to known materials and does not provide information on the mass spectra of unknown molecules. One of the alternative way is to use *de* *novo* techniques, which aim to directly predict the molecular structure based on the input spectrum^11–13^. However, the method often showed low accuracy and limited effectiveness^14^.

To address the coverage issue in the library search-based methods, mass spectrum prediction models utilize either quantum mechanical calculations^15–18^, or machine learning techniques^19^. These methods aim to predict the fragmentation patterns that occur after ionization. Quantum mechanical calculations are computationally inefficient, because they require extensive computations of electronic states.

Recently, a deep learning-based models have been significantly developed in the area of material science and drug development. In particular, Graph Neural Networks (GNNs) have widely been used to predict chemical properties and generate new molecules because molecules can be represented using graph structures, where nodes and edges of a molecule can naturally be defined as atoms and edges, respectively.

Several studies focused on predicting mass spectra using various deep learning models such as GNN and graph transformer^20–24^. J. Wei et al.^20^ proposed the NEIMS model, which utilizes fingerprints to map molecules. They employ MLP layers and a bidirectional prediction mode to model fragments and neutral losses in a mass spectrum. B. Zhang et al.^22^ employ a graph convolutional network (GCN) for mass spectra prediction. They initialize the nodes’ features using concatenated one-hot vectors representing various atom properties such as atom symbol, degree, valence, formal charge, and radical charge, etc. The initial features of edges are also represented using one-hot vectors based on bond type, ring presence, conjugation, and chirality. Multiple GCN layers are applied, and the nodes’ representations are pooled to form a graph representation. An MLP layer is then used to predict the mass spectra.

Other recent papers^23,24^ targeted more complex molecules for motif prediction. A. Young et al.^23^ proposed the MassFormer framework, which is based on the graph transformer for predicting tandem mass spectra. They calculate pairwise attention between node pairs considering the shortest path distance between two nodes, and incorporates averaged edge information along the shortest path. In the work by M. Murphy et al.^24^, they proposed a prediction model that maps an molecular graph to a probability distribution across various molecular formulas using high-resolution mass spectra. This model differs from our approach in that high-resolution mass spectra contain additional information about specific peaks, which can be utilized to infer the molecular formulas associated with those peaks.

Motifs are generally considered as important and frequently occurring substructures, which are one of the main interests as important functional groups and fragments in the field of analytical chemistry^25^. Motif mining is beneficial for various chemical fields and has been widely adopted in many tasks. There are different approaches of motif mining: one is the rule-based approach, where fragmentation rules are defined based on domain knowledge. However, this approach may not effectively cover all possible types of fragments. Alternative approach employs the quantitative analysis of subgraph structures. This approach is inspired from Byte Pair Encoding (BPE)^26^, a widely used technique in Natural Language Processing (NLP) for tokenizing words into subwords. Motifs can be used to improve the model capability for property prediction, drug-gene interaction prediction and molecule generation, in virtue of the strong dependence of a molecule’s properties on its molecular functional groups^27–32^.

In this study, we propose the Motif-based Mass Spectrum prediction Network (MoMS-Net) for prediction of molecular mass spectra with its substructures. Specifically, we chose a heterogeneous graph network as our model framework. In contrast to homogeneous graph networks, heterogeneous graph networks have an advantage in representing diverse features and their relationships derived from various categories. Considering the distinct nature of between molecule and motif graphs in our task, we adopted for a heterogeneous graph network as the base architecture for MoMS-Net.

**Figure 1:**
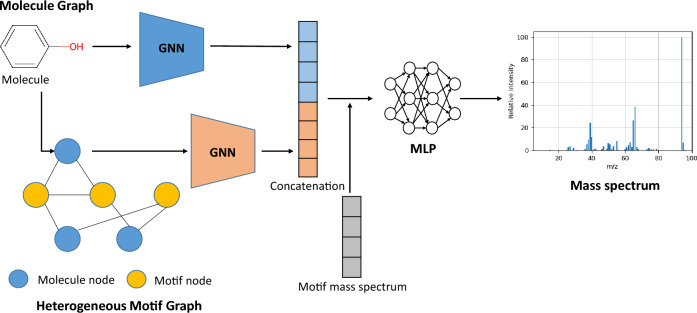
Overall architecture of MoMS-Net. The model consists of two GNN-based encoders, one for molecule graph and the other for heterogeneous motif graphs, respectively. Both types of graph embeddings from each GNN encoder are concatenated to form a single vector, and an MLP layer is finally applied to obtain the mass spectra prediction.

We represent both a molecule and its motifs as a graph form, and apply two types of GNNs as encoders to stably capture the relations between the fragment ions and the associated patterns in the mass spectra. The motif vocabulary is constructed by following the merge-and-update method introduced by Z. Geng et al.^33^. The MoMS-Net model consists of two GNNs: one is for the target molecule graph, and the other is for heterogeneous motif graphs, which handles of all molecules in the dataset and the motifs in the motif vocabulary, as described in Fig. 1. We incorporate the knowledge and characteristics of motifs’ mass spectra as input features, before applying the Multi-Layer Perceptron (MLP) layer to predict a mass spectrum. GNNs struggle to consider long-range dependencies between nodes in the message propagation and pooling operation of neighboring nodes^34–36^. While deep layers are required to utilize information from long-range relations in GNNs for various graph tasks, deep GNNs often struggle to distinguish node features and lead to the oversmoothing problem, which causes a harmful effect on model performance^37–39^. To circumvent the problem, we model the relationship between motif subgraphs at the (sub)graph level rather than a node level, to effectively incorporate long-range dependency relationships. The conventional graph transformer-based models^23,40^ demonstrated fine performance in predicting mass spectra, however, they require a tremendous amount of memory in the training step. In contrast, MoMS-Net requires less memory than the graph transformer-based models. Finally, MoMS-Net achieves the state-of-the-art performance in predicting mass spectra. The main contributions of MoMS-Net are summarized as below.

- MoMS-Net incorporates the relationship between substructures at the graph level, allowing to effectively consider long-range dependencies in molecular graphs. In contrast, conventional GNNs have a difficulty in handling long-range dependency due to updating and pooling mechanism at the node level.
- MoMS-Net achieved the state-of-the-art prediction performance in the public mass spectra benchmark, including the spectrum similarity score and the molecule identification test. In addition, various ablation studies and visualizations validated our model performance.
- MoMS-Net has better scalability and applicability to larger molecules, requiring less memory compared to the previous graph transformer-based models.

## Results

### Overview of the framework

The MoMS-Net model consists of two Graph Neural Networks (GNNs) designed to handle both the molecule graphs and the heterogeneous motif graphs, respectively. The molecule graphs utilizes a 3-layer Graph Convolutional Network (GCN)^41^ with RDkit fingerprints^42^ as input embeddings. On the other hand, the heterogeneous motif graph employs a 3-layer Graph Isomorphic Network (GIN)^43^ with input embeddings based on the relationships between molecules and motifs, motifs and motifs, and molecular weights. Two types of the hidden representations obtained from the last layer of a molecule graph GNN and the heterogeneous motif graph GNN are concatenated into one vector to capture the combined information from both graphs. Additionally, the molecular weight distribution of motifs and selected fragments are utilized to further optimize the hidden representations. Finally, the fully connected layers are applied to the hidden representation in order to predict the mass spectrum. The detailed methods are described in Section 4.

### Spectrum similarity

The results for the cosine similarity of the predicted mass spectra using the NIST dataset are presented in Table 1. MoMS-Net achieves the best performance compared to other models. Specifically, MassFormer outperforms CNN, WLN^44^ and GCN models. In addition, we observed that the performance on the FT-HCD dataset is higher compared to the FT-CID dataset. This can be attributed to the larger amount of data available in the FT-HCD dataset. It is commonly known that transformer-based models can achieve better performance when trained on larger dataset^45^. However, it is noteworthy that MoMS-Net surpasses the performance of MassFormer even in the larger FT-HCD dataset.

**Table 1:** The cosine similarity between predicted and the ground truth mass spectra. MoMS-Net shows the best performance on both the FT-CID and FT-HCD datasets.

	FT-CID	FT-HCD
CNN	$0.356\pm 0.002$	$0.535\pm 0.002$
MassFormer	$0.385\pm 0.005$	$0.573\pm 0.003$
WLN	$0.357\pm 0.001$	$0.569\pm 0.001$
GCN	$0.356\pm 0.001$	$0.565\pm 0.001$
MoMS-Net (Ours)	$\mathbf {0.388\pm 0.002}$	$\mathbf {0.578\pm 0.001 }$

Every result is repeated five times with different random seeds. Significant values are in bold.

The NIST dataset was split into three subsets for the experiments: training (70%), validation (20%), and test (10%), using the Murcko scaffold splitting method^46^. We made predictions for the mass spectra of the molecules in the test set. We then calculated the spectrum similarity between the target (actual) mass spectra and the predicted mass spectra using the cosine similarity score. To ensure a fair comparison, we initially normalized both the target and predicted mass spectra. Then, we computed the cosine similarity score between the normalized vectors. Each result has been obtained by conducting the experiments five times, with distinct random seeds for each run.

### Molecule identification

To evaluate the similarity between spectra in the query and reference sets, we calculate a ranking of spectra in the reference set for each query. The ranks are determined by the degree of similarity and effectively. They also induce a ranking of candidate structures since each spectrum corresponds to a specific molecule. Table 2 provides a summary of the results obtained from this experiment on the metric, top-5%. This metric evaluates whether the true matched candidate is ranked within the top 5% of all candidates. As the number of candidates per query may vary, the top-5% metric is normalized to ensure fair comparison. This metric highlights the model’s ability of accurate identification performance even in a large candidates. The results indicate that our model demonstrates comparable performance with MassFormer and higher than other models. This consistent strong performance of our model suggests that it is one of the best performing models in terms of accurately matching query spectra with the correct molecule. Our model can be utilized for augmenting spectral libraries, which holds promise to address the coverage issue.

**Table 2:** Top-5% scores on the ranking task. MoMS-Net demonstrates outperform in FT-CID dataset, and comparable performance to MassFormer on FT-HCD dataset, respectively.

	FT-CID	FT-HCD
CNN	$0.802\pm 0.008$	$0.778\pm 0.004$
MassFormer	$\mathbf {0.850}\pm 0.016$	$0.830\pm 0.007$
WLN	$0.736\pm 0.011$	$0.812\pm 0.008$
GCN	$0.728\pm 0.0.016$	$0.802\pm 0.008$
MoMS-Net (Ours)	${0.824\pm 0.002}$	$\mathbf {0.840\pm 0.010 }$

Every result is repeated five times with different random seeds. Significant values are in bold.

To address the coverage issue in the library searches, predicting mass spectra is an essential step to enrich the existing database. Following from the previous study, we can employ a candidate ranking experiment introduced by^20,23^. In this experiment, the objective is to accurately associate a query spectrum with the corresponding molecule from a set of candidate spectra. The query set comprises authentic spectra from the test set, which are heldout partitions. The reference set consists of spectra collected from distinct origins: predicted spectra in the heldout partition, and real spectra from the training and validation partitions.

### The effect of motif vocabulary size

**Figure 2:**
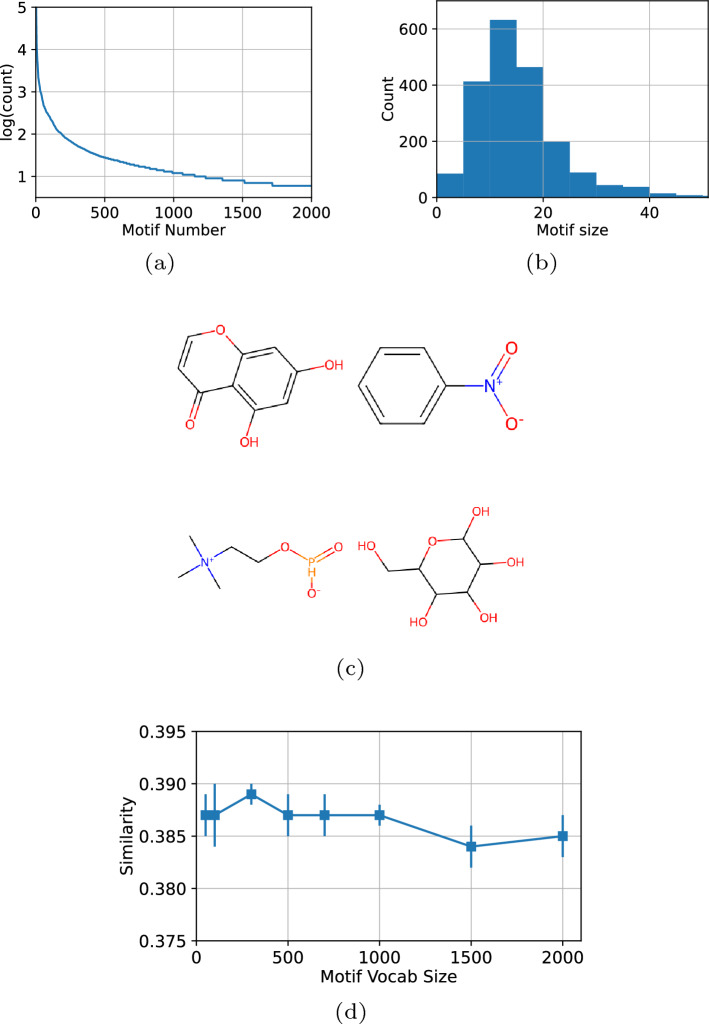
(**a**) Frequency of generated motifs (**b**) The distribution of motif size (**c**) Some examples of large motifs (**d**) Cosine Similarity according to Motif Size. The frequency of motif is decreased exponentially as motif number and most motif has size of 5 to 20 atoms. Data-driven motif generation method can generate large motifs which have various functional groups. The model achieves its best performance when the motif size is adjusted to 300. However, as the motif size surpasses 1000, the performance starts to decline.

We utilized the merge-and-update method to generate motifs from the dataset^33^. The top *K* most frequent subgraphs were chosen as motifs. We examined the resulting motif vocabulary and observed a decreasing exponential trend in the frequency count as the number of motifs increased, as shown in Fig. 2. The most frequent subgraphs are small and stable fragments such as “$\textrm{CC}$”, “$\textrm{CCCC}$”,“$\textrm{CO}$” and benzene ring ($\mathrm {C_6 H_6}$). Our approach allowed for the generation of motifs of various types and sizes, with a higher occurrence of motifs containing 5 to 15 atoms. Fig. 2(c) displays several examples of large motifs with distinct structures. We performed tests with different sizes of motif vocabularies and observed that when the motif size exceeded 1,000, the cosine similarity began to decrease. It can be caused by the inclusion of trivial motifs in the heterogeneous motif graph as the motif vocabulary size increased. From the observation, we set the motif vocabulary size to 300 in all experiments.

### The analysis of predicted spectra

**Figure 3:**
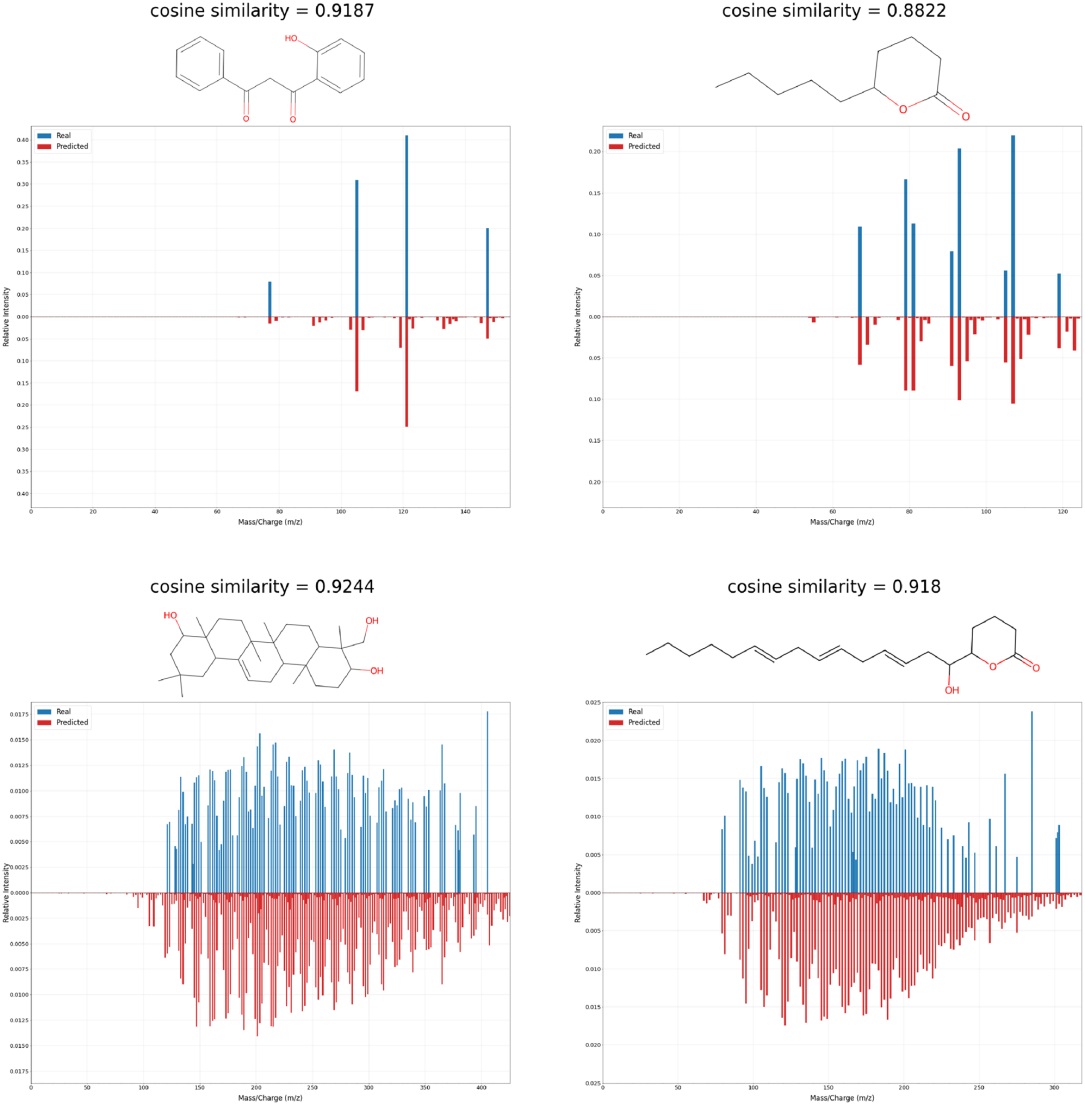
Examples of the real and predicted spectra for the four molecules. The real and predicted spectra exhibit comparable patterns, showcasing similar characteristics for complex molecular structures, irrespective of whether they contain an aromatic ring or not. However, it is noticeable that the predicted spectra display lower intensity, likely due to the presence of smaller peaks.

MoMS-Net demonstrates the capability to accurately predict mass spectra for complex molecules, as shown in Fig. 3. Molecules containing conjugated aromatic rings are known to be highly stable, resulting in a smaller number of peaks in their mass spectra. On the other hand, molecules without aromatic rings tend to exhibit a greater number of peaks. Our model is effective in predicting both aromatic and nonaromatic compounds accurately. However, it should be noted that there is a limitation in terms of the abundances of the main peaks in the predicted mass spectra. Our model tends to generate more smaller peaks, which can result in a decrease in the intensity of the main peak after normalization.

On the top of that, we also visualized the t-SNE plot of the hidden representations in the final layer of MoMS-Net, in Appendix D. We observed that the molecules with higher similarity score tend to more closely related.

### Computational efficiency

In Table 3, the information regarding the number of model parameters and memory allocation is presented. It is observed that all models have a similar order in terms of the number of model parameters, indicating comparable complexity in terms of the model architecture and parameter count. However, we note that MoMS-Net allocates lower memory allocation than MassFormer does. Despite MassFormer having a smaller batch size, it requires a larger amount of memory allocation compared to MoMS-Net. This suggests that MassFormer consumes a significant amount of memory during its execution. On the other hand, MoMS-Net demonstrates better performance while utilizing less memory compared to MassFormer. This efficiency in memory usage allows MoMS-Net to handle larger molecules and proteins effectively.

**Table 3:** The number of parameters and memory allocation. MoMS-Net requires the same memory allocation order as GCN. However, MassFormer requires a larger amount of memory despite having a much smaller batch size.

	# of parameters	Memory allocation(MB)	Batch size
CNN	7.60E+06	718	1024
MassFormer	6.61E+06	1923	128
WLN	5.25E+06	1531	1024
GCN	1.01E+07	1002	1024
MoMS-Net (Ours)	1.81E+07	1409	1024

### Ablation study

The comparison of the prediction performance among GNN encoder architectures is presented in Appendix Table B2. We observed that MoMS-Net with the GCN encoder performs better in mass spectra prediction than MoMS-Net based on the GIN. In the MoMS-Net model, both GIN and GCN are evaluated for the molecular graph, while GIN is specifically used for the heterogeneous motif graph. Interestingly, when GIN is employed instead of GCN for the molecular graph, the MoMS-Net model exhibits similar performance. This finding suggests that both GIN and GCN can effectively capture the structural information present in the molecular graphs and provide valuable insights for mass spectrum prediction by MoMS-Net.

The effect of motif spectra on mass spectrum prediction is evident in Table B2, where a decrease in performance is observed when motif spectra are not considered compared to the MoMS-Net model. This finding indicates that incorporating the information from mass spectra into the motifs has a positive effect on predicting mass spectra accurately. By including the information of mass spectra for each motif, the model gains additional insights into the specific fragmentation patterns associated with different motifs. This allows the model to capture the relationships between the structural features represented by the motifs and the resulting mass spectra more effectively.

We also conducted ablation studies to analyze various model hyperparameters. These included the ratio of the heterogeneous motif graph of molecules, methods for combining heterogeneous features, the size of the hidden dimension, and the size of the training set. The results of these studies are shown in Appendix Figures B1 to B4, respectively.

## Discussion

In this study, we proposed the MoMS-Net model, which incorporates motifs to predict mass spectra from molecular structures. Motifs play an important role in the task of predicting molecular properties, because they are closely related to the functional groups present in the molecule, providing valuable information on the relationships between molecules. The representations of motif graphs inherently need to be distinct from those of molecular graphs because of differing definitions of nodes(edges) in motif graphs and molecule graphs. In addition, a homogeneous graph network cannot explicitly represent the relationship between motif graphs and molecular graphs. In order to effectively capture and learn these inherent heterogeneities, we designed MoMS-Net with a heterogeneous graph network architecture. This allows us to effectively represent and learn both the features of molecular and motif graph representations, originating from fundamentally different sources. We applied the merge-and-update method to generate a motif vocabulary from the dataset to represent various motif sizes and functional groups. We conducted tests with different sizes of motif vocabularies and varying model architectures.

The interpretation of mass spectra can be complicated due to the presence of various functional groups and structural subunits in molecules^47^. Different functional groups, such as oxygen, nitrogen, phosphorus, sulfur, and halogens, have specific fragmentation rules that govern their behavior during mass spectrometry. To address this challenge, we developed a deep learning-based called MoMS-Net, which incorporates the motifs as frequently observed substructures in molecules. These motifs capture important information about the presence and arrangement of functional groups and structural subunits within the molecule. By incorporating these motifs into its architecture, MoMS-Net is able to effectively learn and represent the fragmentation rules associated with different functional groups. This enables the model to accurately predict the mass spectra of molecules. In other words, MoMS-Net demonstrates good performance in predicting how a molecule will fragment in a mass spectrometer based on its structural features and functional groups.

The t-SNE analysis performed on the hidden representation of the final layers in MoMS-Net provides insights into the model’s ability to differentiate molecules based on their cosine similarity between predicted and real mass spectra. In Appendix Figure D, it is observed that molecules with high similarity are well separated from molecules with low similarity in the hidden representation space. This finding indicates that MoMS-Net has the capability to effectively capture and represent molecular features that contribute to the similarity or dissimilarity between molecules. The model’s architecture and training process enable it to learn and encode the structural and chemical information that is relevant for predicting similarity. However, the exact reasons for MoMS-Net’s good predictive performance and the specific features it relies on for distinguishing between similar and dissimilar molecules still require further investigation. Understanding these underlying factors would provide valuable insights into the model’s inner workings and potentially offer opportunities for its improvement. Therefore, we plan to conduct future research to delve deeper into the reasons behind MoMS-Net’s successful predictions. This ongoing work aims to gain a more comprehensive understanding of the model’s strengths and limitations, ultimately enhancing its performance and applicability in various domains related to molecular analysis and characterization.

The motif vocabulary in MoMS-Net is constructed based on the occurrence of motifs in the dataset. These motifs are sorted according to their frequency of occurrence. In the analysis performed on the motif vocabulary, the motifs are categorized into three groups based on their occurrence. The first group consists of motifs that have the highest frequency of occurrence in the dataset. On average, these motifs appear more frequently compared to other motifs in the other two groups. The high occurrence of these motifs suggests that they are common and widely present in the molecules within the dataset. The third group, on the other hand, contains motifs that have lower occurrence compared to the first group. Despite their lower frequency, the third group of motifs exhibit a higher correlation coefficient between cosine similarity and the total number of occurrence for each group. This finding suggests that these less common motifs play a crucial role in the prediction of mass spectra. In other words, the less frequent motifs that show a higher correlation coefficient are indicative of important and informative structural patterns that are specific to certain molecules or functional groups, whereas the most frequent motifs capture common structural features. These rare motifs may contribute significantly to the accurate prediction of mass spectra and provide valuable insights into the fragmentation patterns of specific molecules. This observation highlights the importance of considering both common and less common motifs in the prediction of mass spectra. By incorporating these informative motifs into its predictions, MoMS-Net can capture the structural diversity and unique characteristics of molecules, ultimately leading to improved performance in mass spectrum prediction tasks.

The importance of motifs is further highlighted in the results of incorporating real mass spectra from MoNa (MassBank of North America) in Appendix Table B3. The performance metrics indicate that including real mass spectra in addition to the motif information has a positive impact on mass spectrum prediction compared to using only the motif data. The Mass Spectrum for motif is generated by the weight distribution of molecular ion considering isotope effect, and we utilize the RDKit package to add a few fragment. But some motifs are existing chemicals and have their own mass spectra, which are available in the MoNA dataset. Specifically, we use EI (electron impact) Mass Spectrum, as it does not contain adduct ions and provides a reasonable understanding of fragmentation mechanism from the molecular ion. Out of the 300 motifs in our vocabulary, there are 148 mass spectra. Comparing this method to the previous approach, incorporating the real mass spectra of motifs leads to the performance increase from 0.388 to 0.392. We are unable to utilize this method for all motifs due to the unavailability of mass spectra. However, the results demonstrate that having precise information on mass spectra is beneficial for predicting mass spectra for molecules.

MoMS-Net outperforms other deep learning-based models in predicting mass spectra from molecular structures. It effectively considers long-range dependencies utilizing motifs at the graph level even though GNNs have limitations in considering long-range dependencies. Additionally, our model requires less memory compared to the graph transformer. We found that the real mass spectra of motifs are useful in predicting the mass spectra of molecules, although the predicted mass spectra may contain smaller peaks or noises. Nevertheless, a limitation of this work is that the structures of the majority of target molecules were relatively small and simple. This constraint may contribute to a decrease in performance, particularly in cases involving complex molecules, which should be overcome in future research. In future work, we strive to enhance the initialization method of mass spectra for motifs and incorporate regularization techniques to prevent false peaks. Furthermore, we plan to apply MoMS-Net to larger molecules such as proteins. Moreover, we plan to explore the application of natural language-based features for molecules, which has gained traction across various fields in recent times. Several recent papers^48–51^ employing large-scale NLP techniques have demonstrated promising results in chemistry. Consequently, incorporating language-based motif representation holds potential and may lead to improved performance in the near future.

## Methods

### Dataset

We use the NIST 2020 MS/MS dataset for training and evaluation. NIST dataset is a widely used public dataset due to its extensive coverage and convenient use in the mass spectrum analysis process. Mass spectra depend on the acquisition conditions. We only use spectra from Fourier Transform (FT) instruments because of the large amount of data available, and we consider their collision cell type (CID or HCD). The information regarding the dataset is summarized in Appendix Table A.

### Generation of motif vocabulary

A motif refers to the most frequently observed substructure, and some motifs are correspond to the known functional groups of molecules. To construct a motif vocabulary, we apply the merge-and-update method introduced by A. Young et al.^33^ to identify common patterns from a given dataset *D*. The goal is to learn the top *K* most frequent subgraphs from dataset *D*, where *K* is a hyperparameter. Each molecule in *D* is represented as a graph, $\mathcal {G=(V,E)}$, where atoms and bonds correspond to nodes $(\mathcal {V})$ and edges $(\mathcal {E})$. Initially, we consider each atom from the molecules as a single fragment.

We merge two adjacent fragments, $\mathcal {F}_i$ and $\mathcal {F}_j$, to create a new fragment, $\mathcal {F}_{ij} = \mathcal {F}_i \oplus \mathcal {F}_i$, using a defined operation “$\oplus$”. The merging process involves iteratively updating the merging graphs, $\mathcal {G}_M^{(k)}(\mathcal {V}_M^{(k)}, \mathcal {E}_M^{(k)})$ at the $k^{th}$ iteration ($k = 0, \cdots , K-1$). If the most frequent merged fragment, $\mathcal {F}_{ij}$, is valid, it is added to the motif vocabulary $\{\mathcal {M}\}$. This process is iterated for *K* iterations to construct the motif vocabulary.

### Construction of the heterogeneous motif graph

The heterogeneous motif graph is generated by combining molecule nodes from the molecular dataset and motif nodes from the motif vocabulary. This graph consists of two types of edges connecting the nodes. One type is the molecule-motif edge, and it is defined by the existence of the motif in the molecule. The other type is the motif-motif edge, which means that two motifs share at least one atom. The different weights are assigned based on their types according to Z. Yu et al.^27^, for differentiating edge types. For the molecule-motif edge, the weight is defined by calculating the term frequency-inverse document frequency (TF-IDF) value. For the motif-motif edges, the weight is calculated as the co-occurrence information point-wise mutual information (PMI). So the edge weight $A_{ij}$ between two nodes (*i*, *j*) is represented as

1 $$\begin{aligned} A_{ij}= \left\{ \begin{array} {cc} \text {PMI}_{ij}, &{}\text {if i, j are motifs} \\ \text {TF-IDF}_{ij}, &{}\text {if i or j is a motif} \\ 0, &{}\text {Otherwise} \end{array} \right. \end{aligned}$$

The PMI value is calculated as

2 $$\begin{aligned} \begin{aligned}{}&\text {PMI}_{ij}= \text {log}\frac{p(i,j)}{p(i)p(j)} \\&p(i,j) = \frac{N(i,j)}{M}, p(i)=\frac{N(i)}{M}, p(j)=\frac{N(j)}{M}, \end{aligned} \end{aligned}$$

where *N*(*i*, *j*) is the number of molecules that have motif *i* and motif *j*. *M* is the total number of molecules, and *N*(*i*) is the number of molecules with motif *i*.

3 $$\begin{aligned} \begin{aligned} \text {TF-IDF}_{ij} = C(i)_{j}\left( \text {log}\frac{1+M}{1+N(i)} +1 \right) , \end{aligned} \end{aligned}$$

where $C(i)_{j}$ is the number of frequency that the motif occurs in the molecule *j*.

### Heterogeneous motif graph neural networks

We apply two different GNNs for molecule graphs and heterogeneous motif graph. The molecule graph represents each atom and bond as a node and an edge, respectively. We utilize a 3-layer GCN to update the atom-level representations. To encode the atom and bond features, we employ the Deep Graph Library (DGL) package^52^, which supports embedding them as either one-hot encoding or numerical values. For the heterogeneous motif graph, we employ the other 3-layer Graph Isomorphism Network (GIN). The total number of nodes in the heterogeneous graph is the sum of the number of molecules ($\vert N \vert$) and the size of the motif vocabulary ($\vert V \vert$). The node feature in the heterogeneous motif graph is represented by the occurrence of motifs and molecule weight for the node. To represent the occurrence of motifs in molecules and other motifs, we create a vector of size $\vert V \vert$, where the values indicate motif occurrences. We apply a linear layer and concatenate it with the molecule weight.

A heterogeneous motif consists of all molecule nodes and motif nodes. Since the number of molecules can be large (e.g., 27K for CID and 232K for HCD), computational resource limitations may arise. To tackle this challenge, we employ an edge sampler to decrease the size of the heterogeneous motif graph. We employ a breadth-first algorithm for hop-by-hop sampling from a starting node^27^. We use a 3-hop sampler, denoted as [$s_1,s_2,s_3$], where $s_i$ represents the number of nodes to be sampled. The first-hop neighbors of molecule nodes are motif nodes only. Before applying GINs, we first utilize a 2-layer MLP for input embedding.

### Mass spectra of motif

After obtaining the graph embeddings for the heterogeneous motif graphs, we incorporate additional information from the mass spectra of motif. This is because the fragmentation patterns in mass spectra are associated with the motif structure. We construct the mass spectra of motifs, taking into account the isotope effect of the molecular ion. Additionally, we incorporate a few fragments generated from RDKit software^42^ into the motif mass spectra.

### Objective function

Cosine similarity is commonly used in mass spectrum library search to compare and quantify the similarity between mass spectra^7^. So we choose cosine distance as loss function as Eq. 4.

4 $$\begin{aligned} \textrm{CD}(\textbf{I},\hat{\textbf{I}}) = 1 - \frac{\sum _{k=1}^{M_{max}}{I_{k} \cdot {\hat{I_k}}}}{\Vert \sum _{k=1}^{M_{max}}{I_{k}}^2 \Vert \cdot \Vert \sum _{k=1}^{M_{max}}{\hat{I}_{k}}^2 \Vert } \end{aligned}$$

where $\textbf{I}$ and $\hat{\textbf{I}}$ are vectors of intensities versus m/z for reference and predicted spectrum.

### Evaluation metrics

The mass spectrum is represented as a vector with a length corresponding to the m/z range, along with intensity values. To measure spectrum similarity as the prediction performance, we compute the cosine similarity score between the target and predicted spectra after normalization.

5 $$\begin{aligned} \textrm{Similarity}(\textbf{I},\hat{\textbf{I}}) = \frac{\sum _{k=1}^{M_{max}}{I_{k} \cdot {\hat{I_k}}}}{\Vert \sum _{k=1}^{M_{max}}{I_{k}}^2 \Vert \cdot \Vert \sum _{k=1}^{M_{max}}{\hat{I}_{k}}^2 \Vert }. \end{aligned}$$

### Supplementary Information

Supplementary Information.

## Data Availability

The NIST 2020 MS/MS dataset used and/or analysed during the current study are available from National Institute of Standards and Technology but restrictions apply to the availability of these data, which were licensed under the United States Department of Commerce Copyright, and so are not publicly available. The MoNa dataset used for ablation study is available from the MoNa database at https://mona.fiehnlab.ucdavis.edu/.
