# Supplementary material for: Mass spectra prediction with structural motif-based graph neural networks
Source: Sci Rep. 2024 Jan 16;14:1400. doi: 10.1038/s41598-024-51760-x (PMC10792027; doi:10.1038/s41598-024-51760-x)
Supplement: Supplementary file 1 — Supplementary Information. [file 41598_2024_51760_MOESM1_ESM.pdf]

Mass Spectra Prediction with Structural Motif-based Graph Neural Networks

Jiwon Park[1,2], Jeonghee Jo[3*], Sungroh Yoon[1,4,5*]

[1]Interdisciplinary Program in Artificial Intelligence, Seoul National University, Seoul, 08826, Republic of Korea.
[2]LG Chem, Seoul, 07795, Republic of Korea.
[3]Center for Neuromorphic Engineering, Korea Institute of Science and Technology (KIST), Seoul, 02792, Republic of Korea.
[4]Department of Electrical and Computer Engineering, Seoul National University, Seoul, 08826, Republic of Korea.
[5]Artificial Intelligence Institute, Seoul National University, Seoul, 08826, Republic of Korea.

*Corresponding authors. E-mails: jh.jo2@kist.re.kr; sryoon@snu.ac.kr;
Contributing author: jwpark21@snu.ac.kr;

Keywords: Mass Spectra, GNNs, motif, deep learning, molecule

Appendix A The Dataset

We use the NIST 2020 MS/MS dataset. Table A1 shows the number of spectra and compounds according to their collision cell type (CID or HCD).

Table A1: The statistics of NIST MS/MS dataset.

Collision Type	# Spectra	# Compounds
FT-CID	27,026	18,257
FT-HCD	322,372	19,620

Appendix B Results of Ablation Study

In the Table B2, MoMS-Net(GIN) uses a GIN for a molecule graph and another GIN for a hetergeneous motif graph. MoMS-Net(GCN) uses a GCN for a molecule graph and a GIN for a heterogeneous motif graph. In addition, various aspects are presented: the ratio of heterogeneous motif graphs in molecule graphs (Fig. B1), performance variations due to different methods of motif addition (Fig. B2), performance variations across various hidden dimensions (Fig. B3), and performance variations based on different split ratios (Fig. B4), respectively. Figure B4 displays comparative results (similarity scores) alongside other models including MassFormer, while the other three figures present results from ablation studies.

Table B2: The cosine similarity according to GNN architecture and motif mass Spectra. MoMS-Net demonstrates similar performance regardless of the GNN architectures. Additionally, incorporating motif spectra in MoMS-Net enhances its performance.

	FT-CID	FT-HCD
GIN	0.352 ± 0.004	0.558 ± 0.001
GCN	0.356 ± 0.002	0.565 ± 0.001
MoMS-Net(GIN)	0.389 ± 0.002	0.575 ± 0.001
MoMS-Net(GCN)	0.388 ± 0.002	0.578 ± 0.001
MoMS-Net(w/o motif spectra)	0.383 ± 0.003	0.576 ± 0.001

Table B3: Cosine similarity according to motif spectrum. To use motif spectrum from MoNA dataset shows a higher cosine similarity.

Motif MS	FT-CID
M.W.	0.388 ± 0.002
MoNA	$\mathbf{0.392 \pm 0.001}$

Appendix C Examples of predicted spectra

We compare more predicted mass spectra in Figure C5.

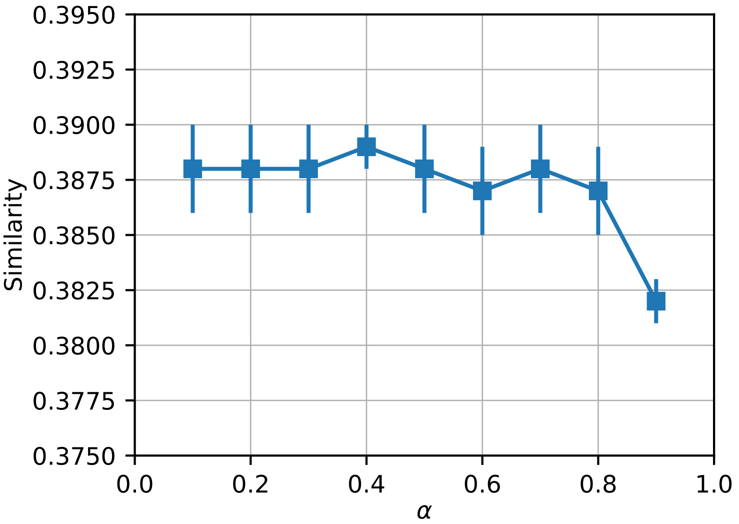

Fig. B1: The ratio α of heterogeneous motif graph to molecule graph. We tested five times for each condition. When α is less than 0.8, the similarity is high, but it decreases when α is larger than 0.8.

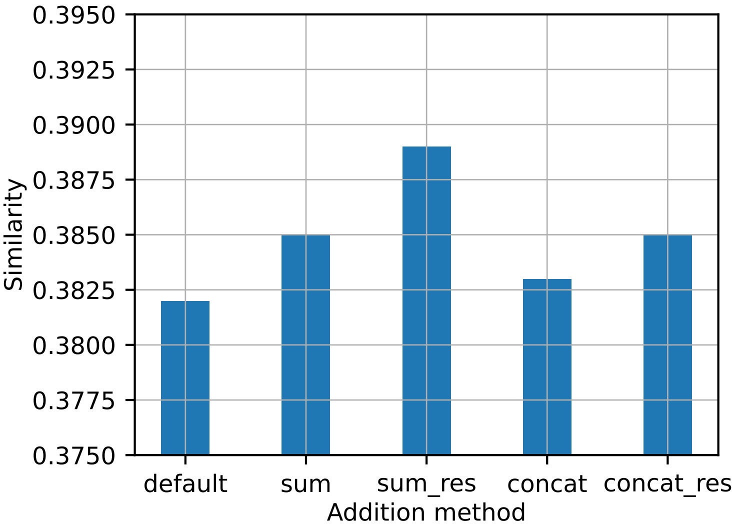

Fig. B2: The various addition methods for motifs. In this context, 'sum' and 'concat' denote the summation and concatenation operations used to combine motif and molecular representation, respectively. Additionally, 'res' indicates a residual connection was additionally applied to the sum or concat operation.

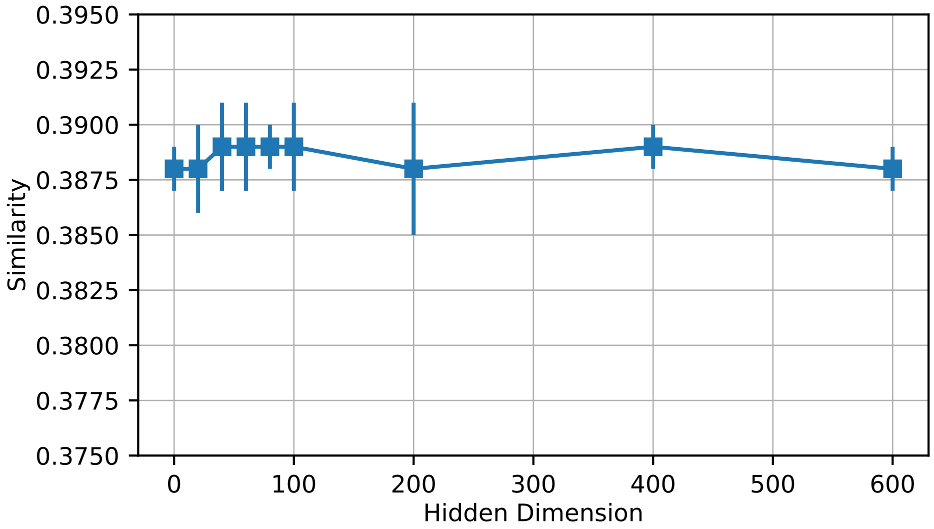

Fig. B3: The hidden dimension size. Two fully-connected layers are applied to embed the input features into the given hidden dimension size. The similarity is not significantly affected by the size of hidden dimension.

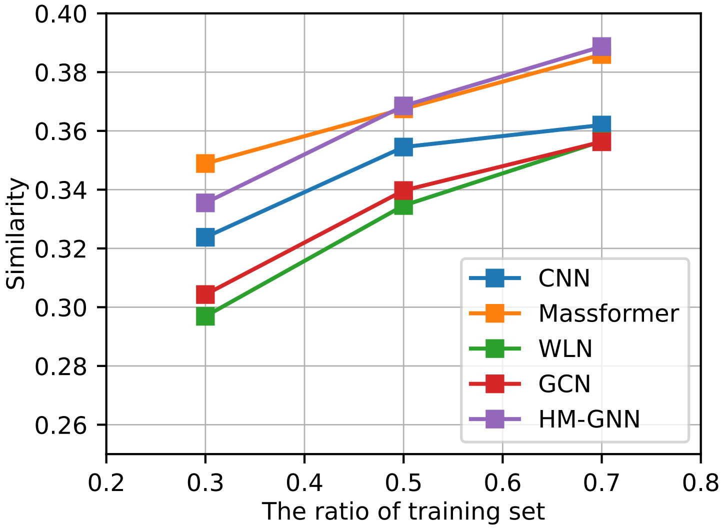

Fig. B4: We tested various models with different split ratios to evaluate model generalizability. MoMS-Net achieved the best performance when the training ratio was set to 0.7, however, Massformer showed slightly better performance when the training size was smaller than 0.7.

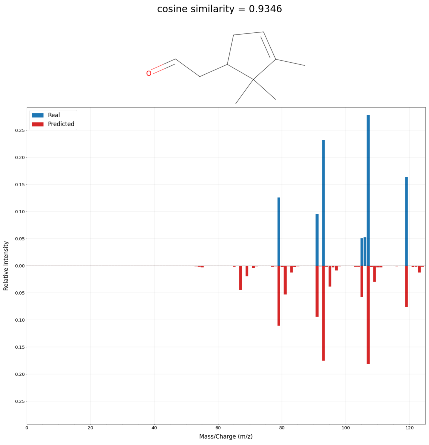
cosine similarity = 0.9346
Real
Predicted
Relative Intensity
Mass/Charge (m/z)

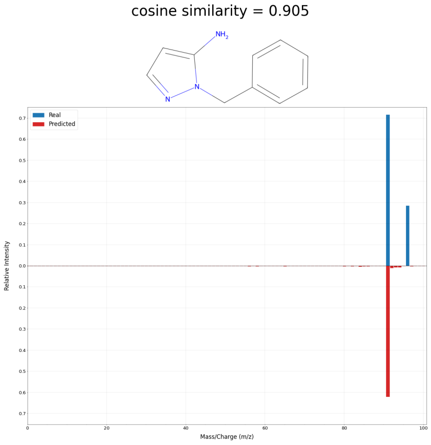
cosine similarity = 0.905
Real
Predicted
Relative Intensity
Mass/Charge (m/z)

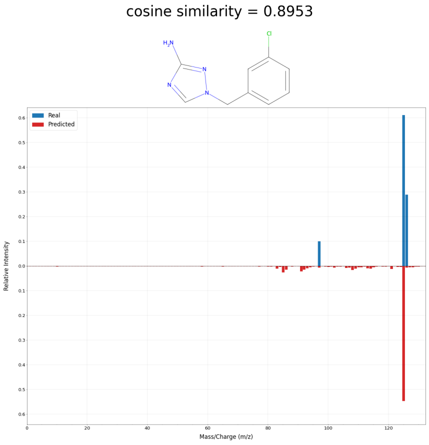
cosine similarity = 0.8953
Real
Predicted
Relative Intensity
Mass/Charge (m/z)

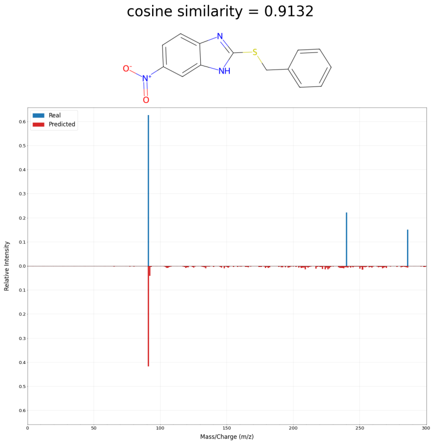
cosine similarity = 0.9132
Real
Predicted
Relative Intensity
Mass/Charge (m/z)

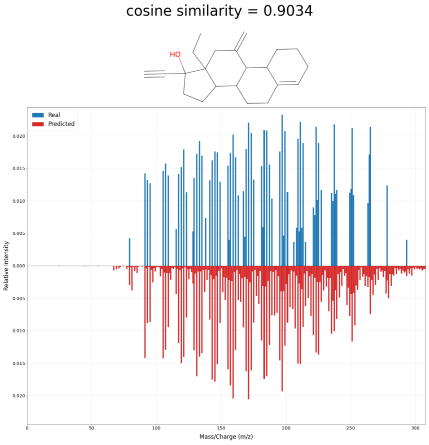
cosine similarity = 0.9034
HO
Real
Predicted
Relative Intensity
Mass/Charge (m/z)

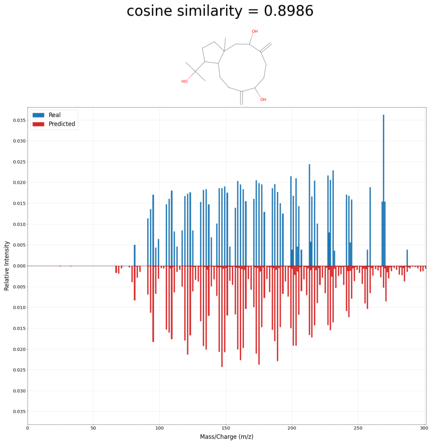
cosine similarity = 0.8986
Real
Predicted
Relative Intensity
Mass/Charge (m/z)

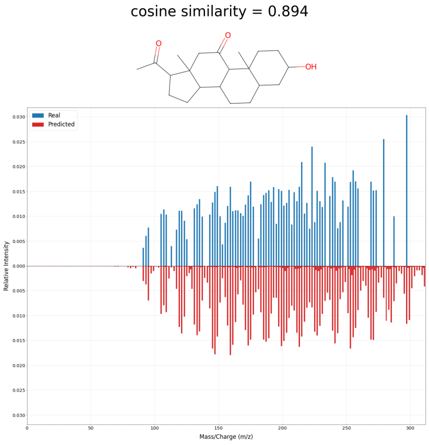
cosine similarity = 0.894
O
O
OH
Real
Predicted
Relative Intensity
Mass/Charge (m/z)

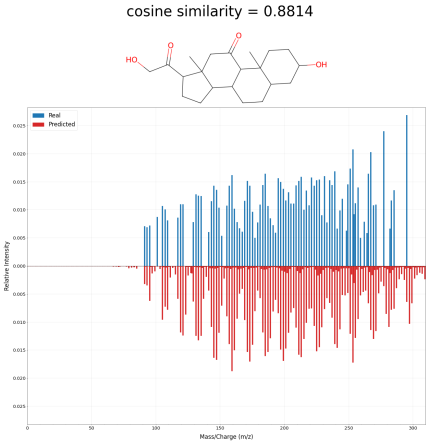
cosine similarity = 0.8814
HO
O
O
OH
Real
Predicted
Relative Intensity
Mass/Charge (m/z)

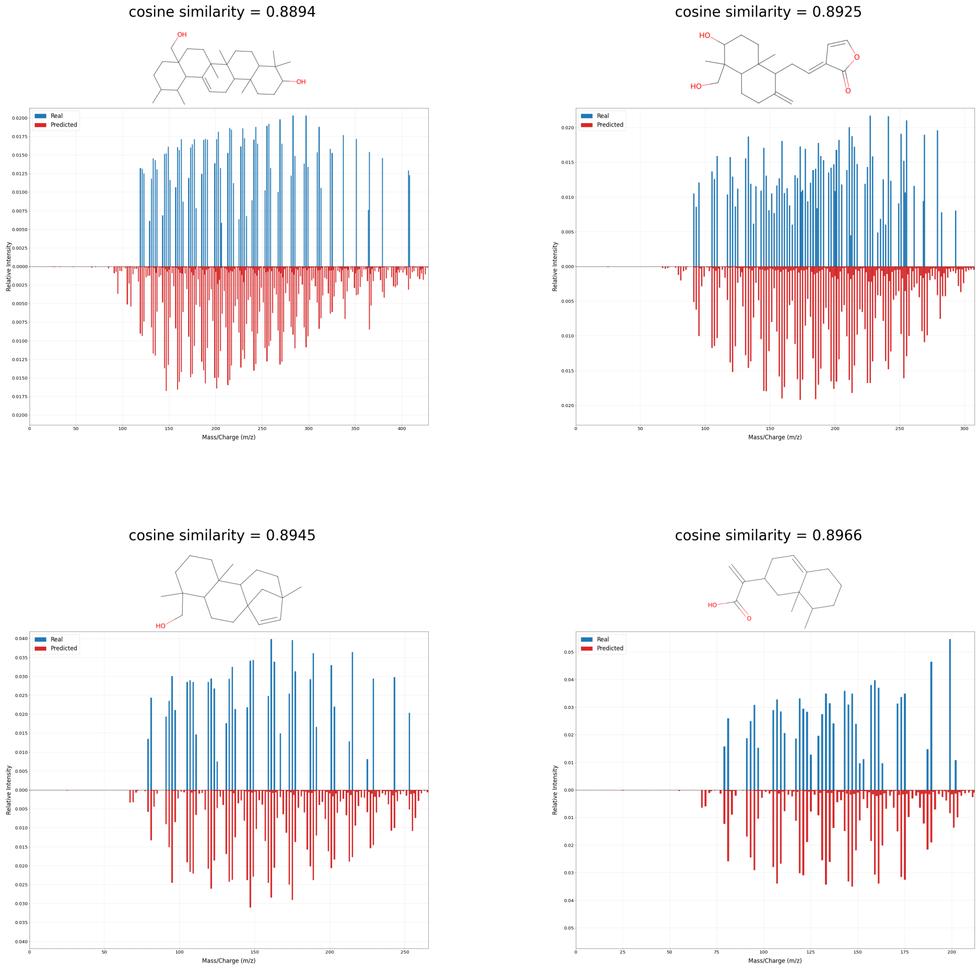

Fig. C5: True and predicted spectra for many sample molecules. Predicted spectra have similar patterns for complex molecular structure but have lower intensity because of many smaller peaks.

Appendix D t-SNE Plot of the Hidden Representation

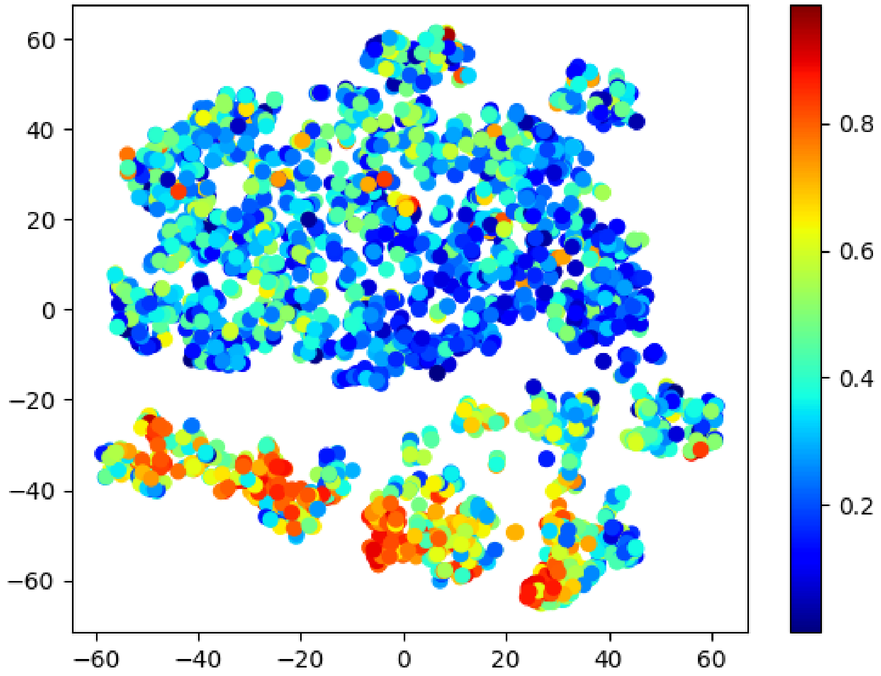

Fig. D6: t-SNE plot of the hidden representation in the final layer. The hidden representation of molecules with higher similarity are well clustered from the others.

Appendix E Correlation Coefficient

The categorization of the motif vocabulary into three groups based on their occurrence and the subsequent calculation of correlation coefficients between cosine similarity for mass prediction and the number of occurrence for motifs in each group have revealed an interesting finding. Specifically, it is observed that the third group, which consists of motifs with the least occurrence, exhibits a higher correlation coefficient compared to the first group. This result indicates that the less frequent motifs in the motif vocabulary, which belong to the third group, play a significant role in the accurate prediction of mass spectra. Despite their lower occurrence, these motifs seem to capture crucial

structural patterns or characteristics that have a strong influence on the prediction of mass spectra.

Table E4: The correlation coefficient between the cosine similarity and the number of occurrence. The less common motif group exhibits a higher correlation with the cosine similarity.

Motif group	Average number of occurrence	Correlation coefficient
1st	22.2	0.133
2nd	8.7	0.307
3rd	5.8	0.358
